# Intestinal Ischemia: US-CT findings correlations

**DOI:** 10.1186/2036-7902-5-S1-S7

**Published:** 2013-07-15

**Authors:** A Reginelli, EA Genovese, S Cappabianca, F Iacobellis, D Berritto, P Fonio, F Coppolino, R Grassi

**Affiliations:** 1 Second University of Naples, Department of Clinical and Experimental Internistic F. Magrassi – A. Lanzara, Naples, Italy; 2 University of Cagliari, Department of Radiology, Cagliari, Italy; 3 University of Turin, Institute of Diagnostic and Interventional Radiology, Turin, Italy; 4 University of Palermo, Department of Radiology, Palermo, Italy

## Abstract

**Background:**

Intestinal ischemia is an abdominal emergency that accounts for approximately 2% of gastrointestinal illnesses. It represents a complex of diseases caused by impaired blood perfusion to the small and/or large bowel including acute arterial mesenteric ischemia (AAMI), acute venous mesenteric ischemia (AVMI), non occlusive mesenteric ischemia (NOMI), ischemia/reperfusion injury (I/R), ischemic colitis (IC). In this study different study methods (US, CT) will be correlated in the detection of mesenteric ischemia imaging findings due to various etiologies.

**Methods:**

Basing on experience of our institutions, over 200 cases of mesenteric ischemia/infarction investigated with both US and CT were evaluated considering, in particular, the following findings: presence/absence of arterial/venous obstruction, bowel wall thickness and enhancement, presence/absence of spastic reflex ileus, hypotonic reflex ileus or paralitic ileus, mural and/or portal/mesenteric pneumatosis, abdominal free fluid, parenchymal ischemia/infarction (liver, kidney, spleen).

**Results:**

To make an early diagnosis useful to ensure a correct therapeutic approach, it is very important to differentiate between occlusive (arterial,venous) and nonocclusive causes (NOMI). The typical findings of each forms of mesenteric ischemia are explained in the text.

**Conclusion:**

At present, the reference diagnostic modality for intestinal ischaemia is contrast-enhanced CT. However, there are some disadvantages associated with these techniques, such as radiation exposure, potential nephrotoxicity and the risk of an allergic reaction to the contrast agents. Thus, not all patients with suspected bowel ischaemia can be subjected to these examinations. Despite its limitations, US could constitutes a good imaging method as first examination in acute settings of suspected mesenteric ischemia.

## Background

Intestinal ischemia is an abdominal emergency that accounts for approximately 2% of gastrointestinal illnesses [1]. It represents a complex of diseases caused by impaired blood perfusion to the small and/or large bowel including acute arterial mesenteric ischemia (AAMI), acute venous mesenteric ischemia (AVMI), non occlusive mesenteric ischemia (NOMI), ischemia/reperfusion injury (I/R), ischemic colitis (IC). The mortality rate is high, ranging between 50–90%, and depends on the etiology, the degree and length of ischemic bowel segments, and the amount of time between the clinical onset of symptoms and the establishment of diagnosis [2-6], so an early diagnosis and treatment are essential to improve the outcome [5,7].

The majority of patients are over the age 60. In case of occlusive etiology, abdominal pain is the most common presenting symptom (94%) and patients usually complain of abdominal pain out of proportion to the abdominal examination. Other symptoms include nausea (56%), vomiting (38%), diarrhea (31%), and tachycardia (31%). In advanced phase, the patient develops peritoneal signs of distention, guarding, rigidity, and hypotension. [8-12]. NOMI is suggested by medical history of systemic hypoperfusion due to major surgery, cardiac impairment, hemorrhage, shock, cirrhosis, sepsis, chronic renal disease, medications, and the use of splanchnic vasoconstrictors [13]

Computed tomography (CT) and ultrasonography (US) are the most commonly used imaging modalities in patients with acute abdomen [14],and even if CT represents the gold standard in the evaluation of patients with AMI, with sensitivity ranging from 82 to 96% and specificity of 94% [4,5,7,15-18], the US, widely available and relatively inexpensive, is more frequently used as first examination in acute settings to rule out other abdominal pathologies.[19,20].

In our series, different method of study (US, CT) will be correlated in the detection of different imaging findings (presence/absence of arterial/venous obstruction, bowel wall thickness and enhancement, presence/absence of spastic reflex ileus, hypotonic reflex ileus or paralitic ileus, mural and/or portal/mesenteric pneumatosis, abdominal free fluid) due to various etiologies of intestinal changes from ischemia and infarction due to mesenteric vessels hypoperfusion or occlusion.

## Methods

Basing on experience of our institutions, over 200 cases of mesenteric ischemia/infarction investigated with both US and CT were evaluated considering, in particular, the following findings: presence/absence of arterial/venous obstruction, bowel wall thickness and enhancement, presence/absence of spastic reflex ileus, hypotonic reflex ileus or paralitic ileus, mural and/or portal/mesenteric pneumatosis, abdominal free fluid, parenchymal ischemia/infarction (liver, kidney, spleen). US was performed with 5.0 MHz convex and linear transducers (Esaote MYLAB™50, Genoa, Italy). US was performed with special attention to the presence/absence of arterial/venous obstruction, bowel wall thicknening (more than 3 mm), presence/absence of spastic reflex ileus, hypotonic reflex ileus (dilation, >2.5 cm, only gas filled) or paralitic ileus (dilation, >2.5 cm, with gas-fluid mixed stasis), mural and/or portal/mesenteric pneumatosis, abdominal free fluid, parenchymal ischemia/infarction (liver, kidney, spleen). Enhanced CT was performed with 64-detector row configuration (VCT, General Electric Healthcare, Milwaukee, Wis, USA). The following techinical parameters were used: in 64-rows CT, effective slice thickness of 3.75 mm for plain acquisition, 1.25 mm in the late arterial phase and 2.5 mm in the portal venous phase; beam pitch of 0.938, reconstruction interval of 0.8mm, tube voltage of 120-140 KVp and reference mAs of 250/700 mA. Automatic tube current modulation was used to minimize the radiation exposure. A standard reconstruction algorithm was used. Patients were instructed not to breath during helical imaging to avoid motion artefacts. All patients received iodinated nonionic contrast material (iopromide, Ultravist 300, Schering, Berlin, Germany) intravenously at a rate of 3.5 mL/s with a power injector. No patient received oral contrast material.

Findings of defects or occlusion of the superior mesenteric artery (SMA) or inferior mesenteric artery (IMA), bowel wall thickening (more than 3 mm in thickness) and enhancement, presence/absence of spastic reflex ileus, hypotonic reflex ileus or paralitic ileus, mural and/or portal/mesenteric pneumatosis, abdominal free fluid, parenchymal ischemia/infarction (liver, kidney, spleen).

## Results and discussion

### Acute arterial mesenteric ischemia

It has been estimated that the majority of cases of intestinal ischemia (65%) are caused by arterial embolism or thrombosis with impairment in the blood flow in the superior mesenteric artery (SMA) distribution affecting all or portions of the small bowel and right colon [13].

#### CT findings

Enhanced CT represents a comprehensive imaging method to evaluate either mesenteric vasculature status or small bowel appearance, both of which have to be evaluated for a diagnosis of ischemia before development of intestinal necrosis and infarction. For a correct interpretation of findings that can be found at CT is necessary to evaluate the vessels; the mesentery and pericolic tissues and the intestinal wall [5] considering that these findings are conditioned by the involved tract (some intestinal segments are more sensitive to ischemic injury) by the typology (varying according to the obstructive mechanisms) and by the time.

**Figure 1:**
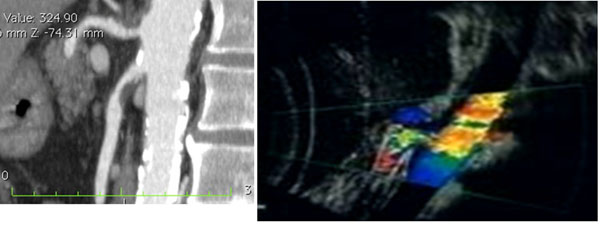
Acute arterial mesenteric ischemia Contrast-enhanced MDCT 2D reconstruction on sagittal plane and US Color Doppler features (b) shows thrombosis with impairment in the blood flow in the superior mesenteric artery (SMA)

**Figure 2:**
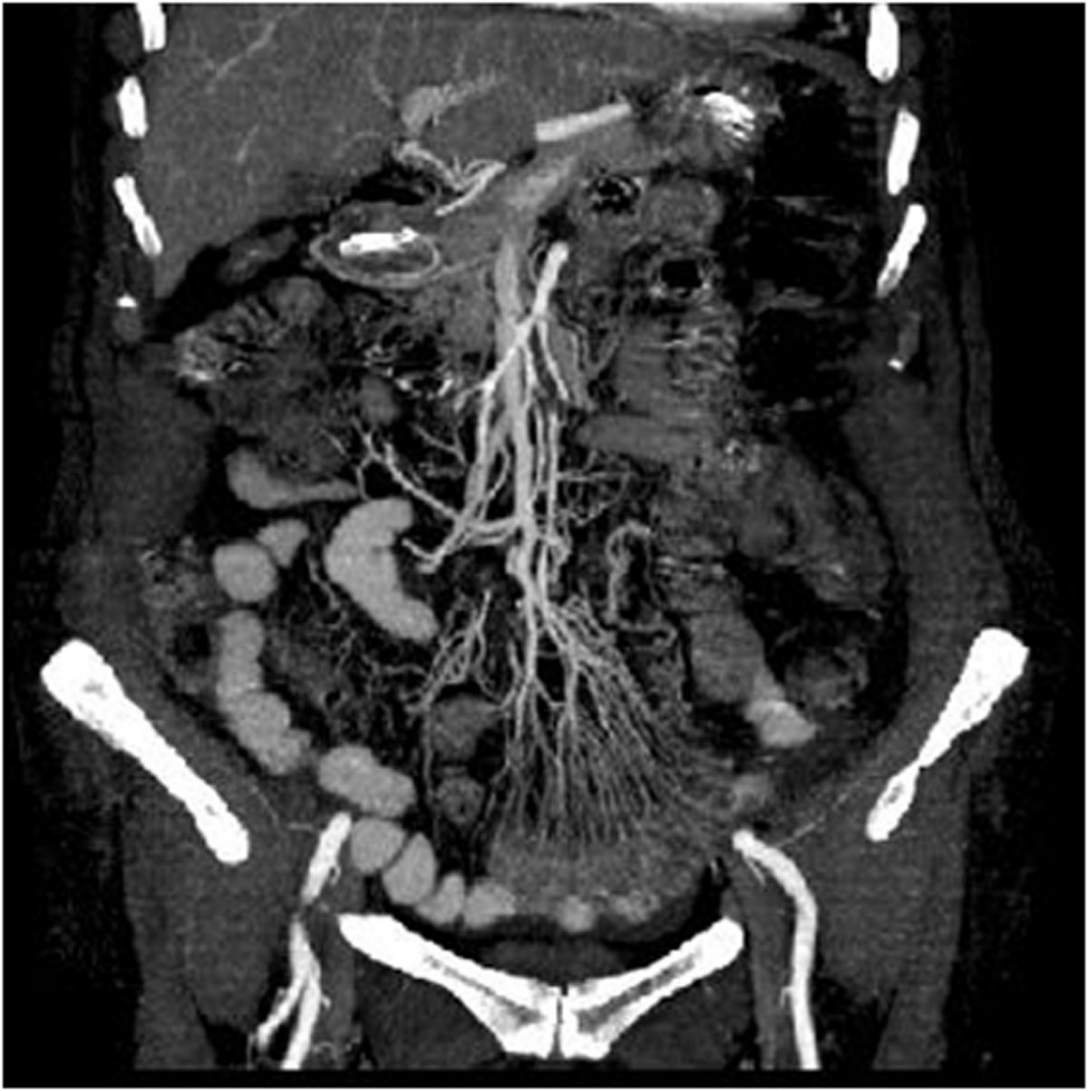
Acute arterial mesenteric ischemia. Contrast-enhanced MDCT 2D reconstruction on coronal plane in early phase: the CT shows the presence of emboli or thrombi as filling defect in the lumen of the artery. If they are small and peripherally localized, the identification can be difficult. The loops of injured small bowel are contracted in consequence of spastic reflex ileus and intestinal wall shows lacking of/poor enhancement. The mesentery is bloodless, due to reduction in caliber of the vessels and apparently in number

Early phase: the CT shows the presence of emboli or thrombi as filling defect in the lumen of the artery [Figure 1a,b]. If they are small and peripherally localized, the identification can be difficult. The loops of injured small bowel are contracted in consequence of spastic reflex ileus and intestinal wall shows lacking of/poor enhancement [Figure 2]. The mesentery is bloodless, due to reduction in caliber of the vessels and apparently in number [1,5,16].

Intermediate phase: blood and fluids are drained by the venous system, not affected by occlusion. The bowel wall become thin, with a typical "paper thin" aspect [14,21], the loops loose the tone, and now are only gas filled so spastic reflex ileus evolves into hypotonic ileus, peritoneal free fluid can be detected too [22].

Late phase: If the causative factor is not removed, the ischemia rapidly evolves into infarction. In the injured loops mount the liquid stasis, air-fluid levels appear and a progression from hypotonic reflex ileus in paralytic ileus can be appreciate [16]. Unfortunately, many patients are diagnosed in this stage because they are overlooked or not identified in previous phases. The wall necrosis lead to parietal, mesenteric, and even portal pneumatosis [23] or perforation with pneumo-peritoneum, retro-pneumo-peritoneum and free fluid in the abdominal cavity [24] due to increased hydrostatic pressure inside the intestinal loops that allows extravasation of plasma and to the peritoneal reaction to the ischemic injury.

#### US findings

In Europe US is frequently performed as primary diagnostic technique for patients with non-specific acute abdominal pain or for patients complaining for intestinal disorders to optimize the use of other imaging techniques [17] or to monitor a pathologic condition that does not require immediate surgery [16]. Sonographic evaluation offers a safe, noninvasive alternative to contrast examinations and, in the clinical suspicion of intestinal infarction, the doppler US could represent a useful modality for the evaluation of severe stenosis in the mesenteric arteries [25-29] and for the evaluation of characteristic intestinal wall changes: in fact relationship between bowel wall changes and the severity of ischemia has been suggested [17]. It should be noted that the assessment potential of this technique is limited if the patient is obese or has an excessive amount of air in the intestinal loops, furthermore, incompliance of patients may limit the accuracy of this imaging modality [30-33]

**Figure 3:**
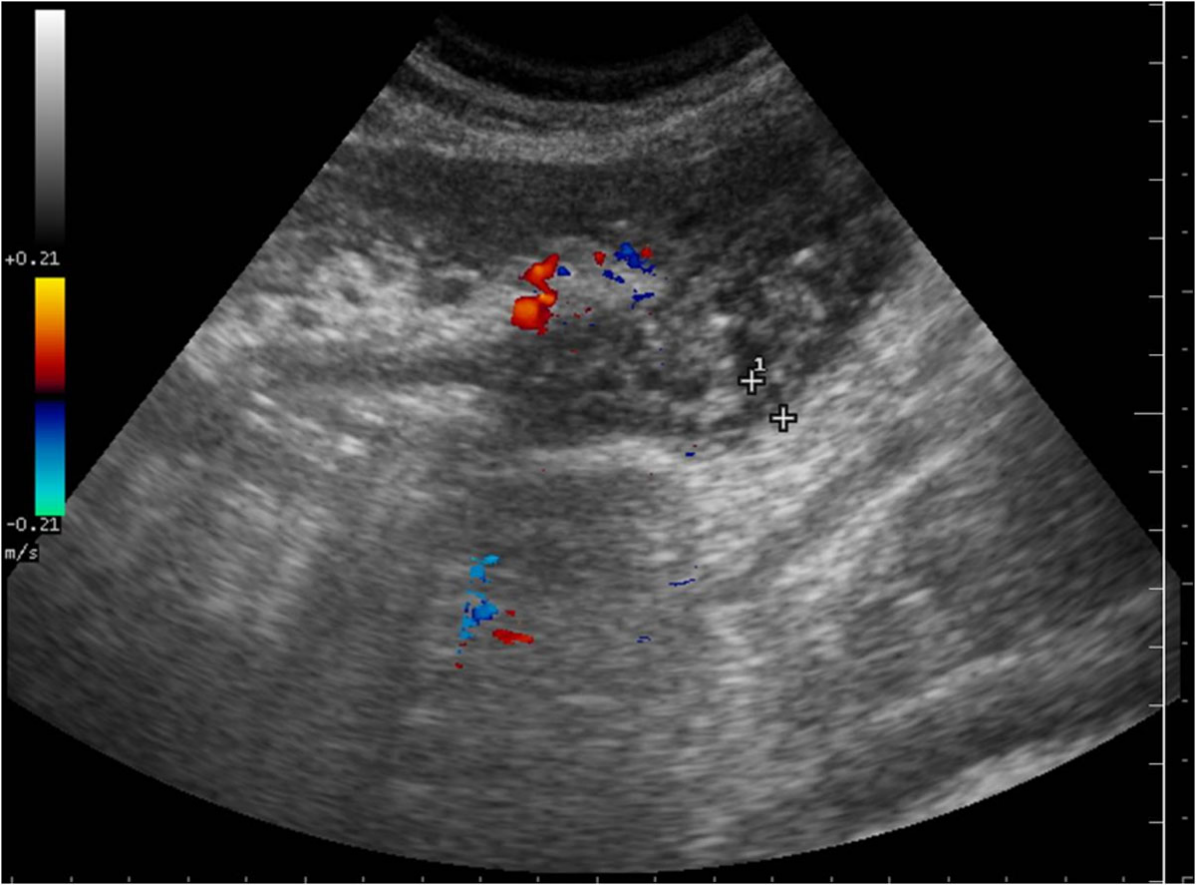
Acute arterial mesenteric ischemia. Sonographic features show increased intraluminal secretions within the involved segments, the spasm of the bowel, the extraluminal fluid and the absent peristalsis

Doppler US can show stenosis, emboli, and thrombosis in the near visible parts of the celiac trunc, the SMA and the IMA. The extend of collateral vessels plays an important role but collaterals cannot be reliably displayed using ultrasound. Colour Doppler and, in some cases, additional echo enhancing agents may be helpful in the evaluation of intestinal wall perfusion and in the identification of the mesenteric vessels. Systolic velocities of more than 250–300 cm/s are sensitive indicators of severe mesenteric arterial stenosis. [34,35]. US may also detect increased intraluminal secretions within the involved segments, the spasm of the bowel, the extraluminal fluid and the absent peristalsis [Figure 3] [13].

The results reported in litterature suggest that in the early phase of bowel ischemia US examinations may show SMA occlusion, and bowel spasm.

In intemediate phase US is not very informative because of an increased amount of gas in the intestinal loops causing large acoustic barrier.

In late phase US may show a fluid-filled lumen, bowel wall thinning, evidence of extraluminal fluid and decreased or absent peristalsis. [16].

### Acute venous mesenteric ischemia

AVMI account for 10% of cases of intestinal ischemia [36]. When there is a complete occlusion of superior mesenteric vein (SMV), the findings are more evident and striking if compared with arterial etiology as it was recently described in an animal experimental model [36]. The SMV occlusion causes impairment in the intestinal vein drainage with consequent vascular engorgement, swelling, and hemorrhage of the bowel wall, with extravasation of fluid from the bowel wall and mesentery into the peritoneal cavity. Venous occlusion causes mucosal edema and punctate hemorrhage that progress to widespread hemorrhages. Progression of the thrombosis and inadequate collateral circulation leads to infarction of the jejunum and the ileum [37].

#### CT findings

**Figure 4:**
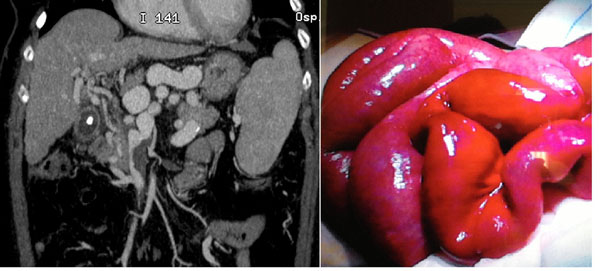
Acute venous mesenteric ischemia Contrast-enhanced MDCT 2D reconstruction on coronal plane in cases of superior mesenteric venous thrombosis in the SMV (a) confirmed at surgery (b).

In cases of superior mesenteric venous thrombosis thrombus may be seen in the SMV at the enhanced CT[Figure 4a,b] [13].

When the venous occlusion persists, there is an increase of intramural blood volume and, consequently, of intravascular hydrostatic pressure with development of interstitial edema, so the imaging findings at this stage of disease are related to mural thickening, intramural hemorrhage, and submucosal edema.[13,16,38]

At CT, can be detected a target appearance of the ischemic bowel with an inner hyperdense ring due to mucosal hypervascularity, hemorrhage, and ulceration; a middle hypodense edematous submucosa; and a normal or slightly thickened muscularis propria.

If the vascular impairment persists, there is a progression to intestinal infarction: the bowel becomes necrotic and peritonitis develop so the CT findings in this phase are represented by mural thickening of the involved segments, peritoneal fluid, and mesenteric engorgement.

In late stage venous thrombosis, absence of mural enhancement, and the presence of fluid and gas may be evident in the mesenteric and portal veins, bowel wall, and sub-peritoneal or peritoneal space.

#### US findings

Ultrasound may show a homogeneously hypoechoic intestinal wall as a result of edema that occurs earlier in the course of disease when compared with SMA compromise.[13,16,38]

**Figure 5:**
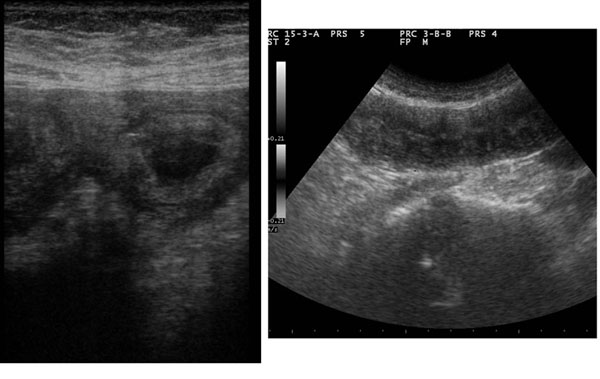
Acute venous mesenteric ischemia Sonographic features show mural thickening with hyperechoic mucosal layers and hypoechoic submucosa attributable to edema of the affected bowel (a). In intermediate phase US examination may reveal increased intraluminal secretions and decreased peristalsis (b).

In initial phase US may reveal thrombus at the SMV origin and mural thickening with hyperechoic mucosal layers and hypoechoic submucosa attributable to edema of the affected bowel [Figure 5a].

In intermediate phase US examination may reveal increased intraluminal secretions and decreased peristalsis [Figure 5b].

In late stage US reveals mural thickening of the involved segment, intramural or intraperitoneal gas, and peritoneal fluid. [13].

### NOMI

NOMI comprises all forms of mesenteric ischemia without occlusion of the mesenteric arteries and accounts for 20–30% of all cases of acute mesenteric ischemia [46-50]

Hypoperfusion of peripheral mesenteric arteries can be caused by different mechanisms and the risk of developing NOMI increases with age. Cardiovascular and drug related factors are risk factors and also various forms of shock, septicemia, dehydration and hypotension following dialysis and heart surgery or major abdominal surgery [47,51]. During low flow states, the entire intestine can be damaged, but the small intestine and the right colon seem to be more sensitive to the states of shock [52-54].

The reduction in blood flow affects both the SMA and IMA, all collateral circulation are therefore ineffective and ischemic lesions and imaging findings have a similar evolution in both the small and in the large intestine.

#### CT findings

**Figure 6:**
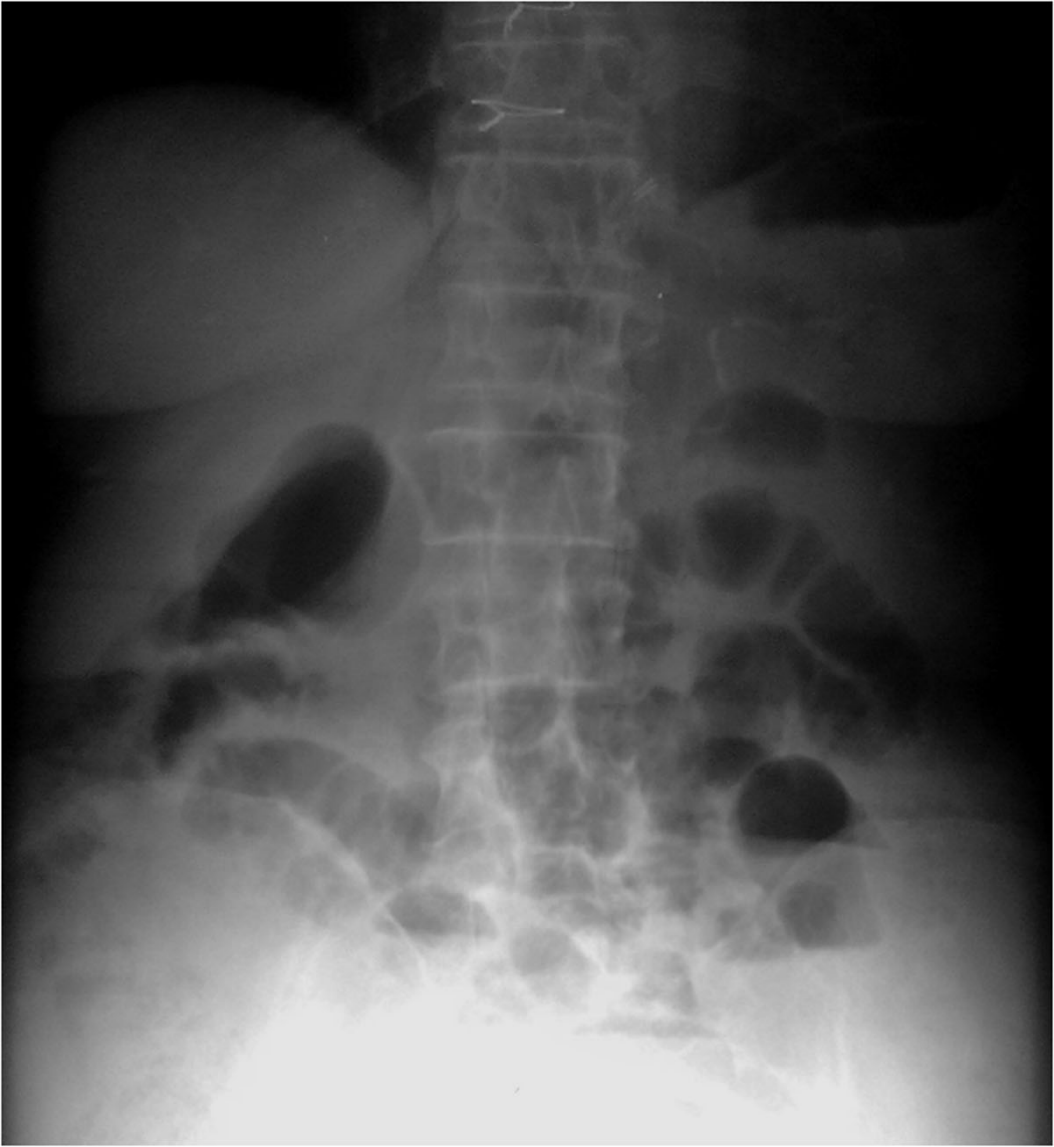
NOMI. Plain abdominal film shows in early phase: ischemia due to vasoconstriction of the splanchnic vessels leading to spastic reflex ileus

Early phase: ischemia due to vasoconstriction of the splanchnic vessels leading to spastic reflex ileus [Figure 6]. The MDCT, unlike the occlusive forms, shows the patency of the mesenteric vessels. Vasoconstriction results in widespread narrowing of the SMA and the mesenteric arcades, with apparent reduction in their number and bloodless mesentery [1,55]. The intestinal wall shows a reduction of enhancement [16].

Intermediate phase: the bowel wall of both small and large bowel appear thinned [55]. If there isn’t reperfusion, the collateral circulation is ineffective and therefore the parietal thinning interested at the same time both the small and the large intestine. All loops are dilated, only gas filled [16,22,46]. the transition from spastic ileus to hypotonic ileus is detected. The mesentery is pale and there also lack of enhancement of the intestinal wall.

If there is a recovery of blood pressure, the intestine is reperfused. Depending on the severity of the damage to the wall of the microcirculation, there is extravasation of plasma and red blood cells with hemorrhagic foci detectable without iv contrast-enhanced CT scans in the form of areas of high attenuation [21]. The edema of the wall thickens the wall that has low attenuation to iv contrast-enhanced MDCT and the typical "target sign" [4,5]. A normal enhancement of the intestinal mucosa is a sign of life [4,21,56].

Late phase: prolonged ischemia, ineffective reperfusion or reperfusion injury, however, can lead to necrosis of trans-mural.

The intestinal segments appear dilated and distended by air-fluid levels, resulting in paralytic ileus.

The absence of enhancement is a sign of ineffective reperfusion which suggests the need for a surgical resection.

#### US findings

**Figure 7:**
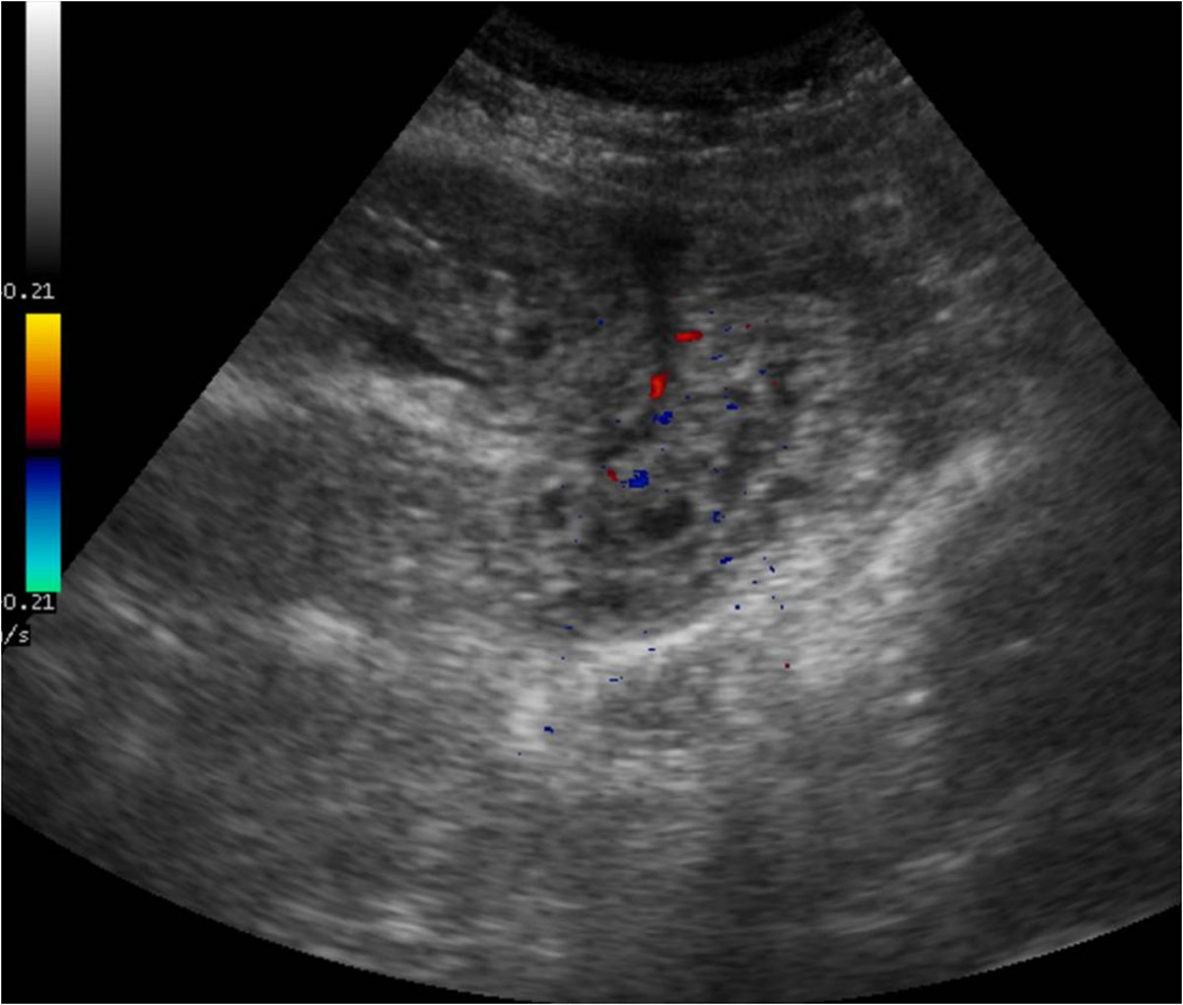
NOMI. US findings. US findings are in the early phase aspecific and poor indicative as thin layer of abdominal free fluid.

US findings are in the early phase aspecific and poor indicative as thin layer of abdominal free fluid, or signs of parenchymal ischemia (not always present); in the intermediate phase the thinning of the bowel wall and the following hypotonic reflex ileus could be observed if there isn’t reperfusion [Figure 7]; if the blood pressure is restored and there is reperfusion damage, bowel wall thickening, hypotonic reflex ileus and gas fluid mixed stasis could be seen. In the late phase, when there is severe necrosis of bowel wall, fluid collections and intramural gas could be found.[46]

### Ischemia/reperfusion injury

To distinguish between mesenteric ischemia with and without reperfusion have a great clinical importance because these conditions have different therapeutic approaches [39,40] and the treatment of an AAMI without reperfusion is significantly different compared to an AAMI with reperfusion [41].

The initial damage caused by ischemia is further worsened by reperfusion [42] with the development of reactive oxygen species, responsible for the reperfusion injury causing tissue injury, altering eicosanoid metabolism, and activating neutrophils and complement [8,43]. Consequently, many cases of intestinal I/R develop into shock, multiple organ failure, and death[8,14,44,45]

#### CT findings

When reperfusion occurs, the findings are very similar to those detected in venous ischemia, [36]

The reperfused intestine may have a different pattern [21], depending on degree of microvascular wall damage, blood plasma, contrast medium, or red blood cells may extravasate through the disrupted vascular wall and mucosa, causing considerable bowel wall thickening and bloody fluid filling of the bowel lumen [16,21].

The entity and extension of damage are related with the duration and degree of ischemia and may even progress to the necrosis of the entire wall.

#### US findings

As consequence of reperfusion, US may show fluid-filled lumen, bowel wall thickening, evidence of some extraluminal fluid and decreased peristalsis. The intestinal mucosa may remain viable if the reperfusion is prompt enough; otherwise, it becomes infarcted and necrotic [21]

### Ischemic Colitis

Ischemic colitis (IC) is considered the most frequent form of intestinal ischemia and the second most frequent cause of lower gastrointestinal

bleeding[8]. It represents the consequence of an acute or, more commonly, chronic decrease or blockage in the colonic blood supply, which may be either occlusive or non- occlusive in origin. Hypertension, diabetes mellitus, ischemic heart disease, congestive heart disease, age and hyperlipidemia are known risk factors. Another risk factor is renal failure. [57-59]

#### CT findings

CT can suggest diagnosis and location of injury and can exclude other serious medical conditions, narrowing the differential diagnosis possibilities [57].

IC generally results in alteration of wall thickness, which in a non-collapsed loop, should measure no more than 3 mm [60]

in the early phase no defects or occlusion of the SMA or IMA are found if IC is caused by NOMI and signs of parenchymal ischemia could be detected.

If IC is due to IMA occlusion, enhanced CT allows to detect the thrombus/embolus; in both cases the presence of pericolic fluid is usually found and in a good percentage peritoneal free fluid is also present. In IMA occlusion the injured colonic wall appeared uniformly thickened with target configuration after contrast medium administration due to reperfusion damage following the restored IMA patency, or the blood perfusion from Riolano’s arcades.

In the intermediate phase: in IC due to NOMI in which reperfusion is not effective, the colonic wall remains thinned and the wall enhancement is compromised. In IC due to IMA occlusion the injured colonic wall appears uniformly thickened with target configuration after contrast medium administration due to reperfusion damage following the restored IMA patency, or the blood perfusion from Riolano’s arcades.

In the late phase: if the reperfusion is effective, a progressive improvement is observed with resorption of free fluid and restoration of the physiological wall appearance. if the reperfusion is not effective there is progression to the bowel necrosis with findings similar to those depicted above with increase of pericolic and peritoneal free fluid, lack of enhancement in the injured wall and in late stages pneumatosis.

#### US findings

It is a sensitive technique for the early detection of changes in the colonic wall caused by CI and can lead to diagnosis in an appropriate clinical context [57].

US could be useful in the evaluation of location and length of the injured colonic segment, and could also detect the wall thickening and stratification, the abnormal ecogenicity of pericolic fat and the peritoneal fluid [57] .

The US with color Doppler can be useful in differentiating between wall thickening from inflammatory or ischemic disease and in identifying patients who will develop necrosis [16,57]

The limitations of this method are related to the operator-dependent quality, the overlying bowel gas and poor sensitivity for low flow vessel disease.

False negatives can be related from tests carried out in the very early stages of IC in which the imaging findings may be normal. The IC with wall thinning could not be identified to the US, although this eventuality is more frequent in cases of acute mesenteric ischemia. Similarly, the pneumatosis intestinalis, finding late and prognostically negative, easily identifiable in CT, is hardly repertabile to the US. [57]

## Conclusion

At present, the reference diagnostic modality for intestinal ischaemia is contrast-enhanced CT. [61] However, there are some disadvantages associated with these techniques, such as radiation exposure, potential nephrotoxicity and the risk of an allergic reaction to the contrast agents. Thus, not all patients with suspected bowel ischaemia can be subjected to these examinations.[62] Despite its limitations, US could constitutes a good imaging method as first examination in acute settings of suspected mesenteric ischemia[63,64].

To make an early diagnosis useful to ensure a correct therapeutic approach, it is very important to define if the vascular impairment involves the superior or the inferior mesenteric vessels and if the etiology is occlusive (arterial, venous) or non occlusive (NOMI), distinguishing between acute arterial mesenteric ischemia (AAMI), acute venous mesenteric ischemia (AVMI), non occlusive mesenteric ischemia (NOMI), ischemia/reperfusion injury (I/R), ischemic colitis (IC). Acute mesenteric ischemia due to occlusion needs an operative treatment while NOMI can be treated non-operatively unless there is evidence of gangrenous bowel [8].

## Competing interests

The authors declare that they have no competing interests.

## Declarations

This article has been published as part of *Critical Ultrasound Journal* Volume 5 Supplement 1, 2013: Topics in emergency abdominal ultrasonography. The full contents of the supplement are available online at http://www.criticalultrasoundjournal.com/supplements/5/S1. Publication of this supplement has been funded by the University of Molise, University of Siena, University of Cagliari, University of Ferrara and University of Turin.
